# Acceptability and design preferences of supervised injection services among people who inject drugs in a mid-sized Canadian City

**DOI:** 10.1186/s12954-017-0174-x

**Published:** 2017-07-14

**Authors:** Sanjana Mitra, Beth Rachlis, Ayden Scheim, Geoff Bardwell, Sean B. Rourke, Thomas Kerr

**Affiliations:** 1 The Ontario HIV Treatment Network, 1300 Yonge Street, Suite 600, Toronto, ON M4T 1X3 Canada; 2 Division of Clinical Public Health, Dalla Lana School of Public Health, University of Toronto, 155 College Street, 6th floor, Toronto, ON M5T 3M7 Canada; 3 Department of Epidemiology and Biostatistics, The University of Western Ontario, K201 Kresge Building, London, ON N6A 5C1 Canada; 4 BC Centre on Substance Use, St. Paul’s Hospital, 608-1081 Burrard Street, Vancouver, BC V6Z 1Y6 Canada; 5 Centre for Urban Health Solutions, Li Ka Shing Knowledge Institute of St. Michael’s Hospital, 209 Victoria Street, Toronto, ON M5B 1T8 Canada; 6 BC Centre for Excellence in HIV/AIDS, St. Paul’s Hospital, 608-1081 Burrard Street, Vancouver, V6Z 1Y6 BC Canada; 7 Department of Medicine, University of British Columbia, St. Paul’s Hospital, 608-1081 Burrard Street, Vancouver, BC V6Z 1Y6 Canada

**Keywords:** Supervised injection services, Supervised consumption facilities, Feasibility research, People who inject drugs

## Abstract

**Background:**

Supervised injection services (SIS) have been shown to reduce the public- and individual-level harms associated with injection drug use. While SIS feasibility research has been conducted in large urban centres, little is known about the acceptability of these services among people who inject drugs (PWID) in mid-sized cities. We assessed the prevalence and correlates of willingness to use SIS as well as design and operational preferences among PWID in London, Canada.

**Methods:**

Between March and April 2016, peer research associates administered a cross-sectional survey to PWID in London. Socio-demographic characteristics, drug-use patterns, and behaviours associated with willingness to use SIS were estimated using bivariable and multivariable logistic regression models. Chi-square tests were used to compare characteristics with expected frequency of SIS use among those willing to use SIS. Design and operational preferences are also described.

**Results:**

Of 197 PWID included in this analysis (median age, 39; interquartile range (IQR), 33–50; 38% female), 170 (86%) reported willingness to use SIS. In multivariable analyses, being female (adjusted odds ratio (AOR) 0.29; 95% confidence interval (CI) 0.11–0.75) was negatively associated with willingness to use, while public injecting in the last 6 months (AOR 2.76; 95% CI 1.00–7.62) was positively associated with willingness to use. Participants living in unstable housing, those injecting in public, and those injecting opioids and crystal methamphetamine daily reported higher expected frequency of SIS use (*p <* 0.05). A majority preferred private cubicles for injecting spaces and daytime operational hours, while just under half preferred PWID involved in service operations.

**Conclusions:**

High levels of willingness to use SIS were found among PWID in this setting, suggesting that these services may play a role in addressing the harms associated with injection drug use. To maximize the uptake of SIS, programme planners and policy makers should consider the effects of gender and views of PWID regarding SIS design and operational preferences.

## Background

Injection drug use is associated with a wide range of health and social harms, including endocarditis [1], soft tissue infections [2], human immunodeficiency virus (HIV) [3, 4], hepatitis C (HCV) [5], and overdose [6]. Aside from the individual-level harms experienced among people who use injection drugs (PWID), at the community level, injection drug use in public spaces contributes to the improper disposal of injection-related litter and is perceived as a public nuisance [7]. Likewise, costs due to injection drug use-related infections take a financial toll on the health care system [8–10].

To address the harms associated with injection drug use, supervised injection services (SIS), also called safer injecting facilities or supervised injection sites, have been implemented in many settings globally, with more than 90 established services in Western Europe, Australia, and Canada [11, 12]. SIS are health services that offer a safe and hygienic environment where people can inject previously obtained illicit substances under the supervision of nurses or other trained health care staff [13, 14]. In addition to allowing people to inject drugs in a low-risk environment, SIS provide clients with access to sterile injecting equipment, connect people to basic medical care, and provide referrals to other health and social services, including treatment for addiction [14, 15].

Previous research has established that SIS have positive impacts on the communities in which they are located, reducing the health and social harms associated with injection drug use. Rigorous evaluation of Insite, North America’s first legally sanctioned supervised injection site located in Vancouver, Canada, demonstrates that SIS reduce the risk of HIV transmission [16, 17] and fatal overdose [18] and increase the uptake of medical care and addiction treatment [19, 20]. The service has been shown to reduce the extent of public injecting and the number of discarded syringes and other injection-related litter in public spaces [7, 21]. Evaluations of the services from Australia have also demonstrated a reduced number of overdose-related ambulance callouts in the neighbourhoods surrounding the services [22].

SIS feasibility research has been conducted in various settings to inform the implementation of SIS and establish the acceptability and willingness to use such services among PWID [23–26]. Importantly, consistent with other literature suggesting that intention is a reasonable predictor of health-related behaviour, intention to use SIS has been shown to predict actual use once SIS are established. DeBeck et al. [27] demonstrated that initial willingness to use a safe injection facility (SIF) among PWID was independently associated with subsequent attendance at a Vancouver-based SIF, even after adjusting for other determinants of willingness to use [27]. While SIS feasibility research has been conducted in large urban centres in North America including San Francisco [23], Vancouver [25, 26], Toronto, and Ottawa [24], little is known about the acceptability and design preferences of SIS among people who inject drugs in mid-sized cities.

Despite often receiving fewer resources, at present many mid-size cities are contending with the same issues related to injection drug use as large urban centres. This is no more evident as in the present opioid and overdose crises spanning across small- to mid-sized North American cities [28]. However, there are some differences between mid- and large-sized cities with respect to barriers of SIS implementation. Existing literature on harm reduction and injection drug use indicates that PWID living in smaller and remote communities often experience limited access to formalized networks of harm reduction and social services, lack anonymity when accessing services [29], and experience inconsistent access to transportation [30]. Social and cultural norms of smaller communities can also make it challenging for PWID to access services. In contrast to large-size cities, smaller communities often face more rigid norms of individualism, self-sufficiency, and conservatism and can lack liberal attitudes toward harm reduction and injection drug use [30, 31]. Subsequent experiences of stigma can lead to the desire of keeping one’s substance use hidden or lead to social isolation, making it difficult for PWID to seek help [30]. Similar challenges may also constrain efforts to implement and promote uptake of SIS and other harm reduction services in mid-sized cities.

London, Canada, is a mid-sized city located in southwestern Ontario, with a population of approximately 370,000 [32]. When compared to other cities similar in size, London bears a disproportionately high burden of injection drug use [33]. The Public Health Agency of Canada estimates that PWID from London experience considerably higher rates of non-prescription opioid injection (69–76%), borrowing and loaning of needles (20 and 27%, respectively), and HCV (79%) compared to national averages [34]. In 2015, London experienced an outbreak of new HIV diagnoses with PWID accounting for two thirds of diagnoses, compared to 12% reported provincially [35]. The harms associated with injection drug use also burden local health services with Emergency Medical Services responding to 603 overdoses in 2013, and rates of opioid-related emergency department visits are 1.5 times higher than the provincial average [34].

Despite a wide range of programming and services available to PWID in London, including naloxone and needle distribution programmes, addictions treatment, street outreach, and supportive housing, problems due to injection drug use persist [34–36]. The potential role and acceptability of SIS in London, however, remains unknown. Therefore, we assessed the prevalence and correlates of willingness to use SIS among PWID in London, Canada, and describe the design and operational preferences among PWID who expressed willingness to use SIS.

## Methods

Survey data were collected from the Ontario Integrated Supervised Injection Services Feasibility Study conducted in London, Canada [37]. Between March and April 2016, the research team worked with three peer research associates (PRAs) who administered a cross-sectional quantitative survey to PWID. PWID who were 18 or older and injected drugs in the last 6 months were eligible for participation. Participants were recruited through city-wide peer outreach efforts, word of mouth, and recruitment flyers posted at local health and social service agencies. Potential participants were then invited for appointment or drop-in interview sessions at three sites. The survey, which was programmed on electronic tablets and took approximately 45 min to complete, collected data on socio-demographic characteristics, drug-using behaviours and related harms, access to health services, willingness to use SIS, and SIS design preferences. The questionnaire was adapted from previous supervised injection services feasibility studies [25]. All participants were provided a $25 honorarium and provided written informed consent.

The study was supported by the Ontario HIV Treatment Network in partnership with the Regional HIV/AIDS Connection, a local AIDS Service Organization, and was guided by an Advisory Committee, composed of local health care and social service providers, and other stakeholders. Ethics approval was obtained from the University of Toronto and the University of British Columbia’s research ethics boards.

### Measures and outcomes

Our primary outcome was willingness to use SIS. Responses were categorized into yes (i.e. those willing to use SIS) and maybe/no (i.e. those who may be willing or not willing to use SIS). Participants were also asked “If a SIS was established in a location convenient to you, how often would you use it?” with response options that included: always (100% of the time), usually (over 75% of the time), sometimes (between 25 and 75% of the time), occasionally (less than 25% of the time), and never. Responses were categorized into always or usually and sometime or occasionally (i.e. high and low expected frequency of SIS use, respectively, defined as always/usually and sometimes/occasionally).

Socio-demographic variables considered for analysis included age (in years), gender (female versus male), ethnicity (White versus Indigenous/Persons of colour), housing status (homeless or unstably housed versus living alone, with a partner, or with family) and involvement in sex work in the past 6 months (yes versus no). Drug-using behaviours were assessed for the past 6 months and included: any public injecting (yes versus no), any injecting alone (yes versus no), any help needed during injecting (yes versus no), any syringe sharing (categorized as borrowing or loaning; yes versus no), and daily opioid injecting and daily crystal methamphetamine injecting (both defined as daily versus less than daily or never). Lifetime history of drug overdose (yes versus no) and history of drug treatment (including past use of one or a combination of the following: detox programmes, opioid substitution therapy, addictions case management, drug court, residential drug treatment and outpatient counselling; yes versus no) were also explored.

Data were also collected on SIS design preferences, willingness to use an integrated service, willingness to walk or bus to SIS, preferred set-up for injecting space, hours of operation, involvement of PWID in service operation, and important amenities for SIS.

### Data analysis

We used descriptive statistics including proportions for categorical variables and report the median (and interquartile range) for age as a continuous variable. Logistic regression was used to model socio-demographic and drug-using behaviours associated with willingness to use SIS. To adjust for potential confounding, all variables were entered into a multivariable logistic model. Using backwards selection, the least significant variable was dropped from the multivariable model, unless dropping it changed the statistical significance of other variables. Reduced models were continually refit until the all variables were either significant (*p <* 0.05) or were considered potential confounders. We explored the expected frequency of SIS use (high versus low frequency) by socio-demographic and drug-using behaviour using chi-square tests or Fishers exact tests (when appropriate). The frequency of design preferences and important amenities for SIS are reported. All analyses were conducted in SAS 9.4 [38].

## Results

In total, of 199 participants who were interviewed, 197 (99%) provided complete data on willingness to use SIS (Table 1). Seventy-five (38%) were female, the median age was 39 years (interquartile range (IQR) 33–50), 73% identified as White, and 139 (72%) reported public injecting in the past 6 months.

**Table 1:** Demographic, drug use characteristics, and treatment history characteristics associated with willingness to use SIS among PWID

Characteristic	Total sample (*n =* 197)	Willingness to use SIS		Unadjusted OR (95% CI)	Adjusted OR (95% CI)
		Yes (*n =* 170) *n* (%)	No or maybe (*n =* 27) *n* (%)		
Age, year					
Median (IQR)	39 (33–50)	39 (32–50)	40 (35–48)	0.99 (0.95–1.03)	1.00 (0.96–1.05)
Gender					
Female	75 (38.1)	57 (76.0)	18 (24.0)	0.25* (0.11–0.60)	0.29* (0.11–0.75)
Male	122 (61.9)	113 (92.6)	9 (7.4)		
Ethnicity					
White	139 (72.8)	120 (86.3)	19 (13.7)	0.98 (0.39–2.50)	0.63 (0.22–1.77)
Other	52 (27.2)	45 (86.5)	7 (13.5)		
Housing					
Unstable	114 (57.9)	103 (90.3)	11 (9.7)	2.24 (0.97–5.11)	1.42 (0.53–3.80)
Stable	83 (42.1)	67 (80.7)	16 (19.3)		
Sex work					
Yes	38 (19.3)	33 (86.8)	5 (13.2)	1.06 (0.37–3.01)	–
No	159 (80.7)	137 (86.2)	22 (13.8)		
Any public injecting^a^					
Yes	139 (71.9)	127 (91.4)	12 (8.6)	3.61* (1.55–8.44)	2.76* (1.00–7.62)
No	55 (28.4)	41 (74.5)	14 (25.5)		
Any injecting alone^a^					
Yes	172 (87.3)	151 (87.8)	21 (12.2)	2.27 (0.82–6.33)	1.68 (0.56–5.10)
No	25 (12.7)	19 (76.0)	6 (24)		
Any help injecting^a^					–
Yes	63 (32.0)	56 (88.9)	7 (11.0)	1.40 (0.56–3.52)	
No	134 (68.0)	114 (85.1)	20 (14.9)		
Syringe sharing^a^					–
Yes	44 (22.4)	39 (88.6)	5 (11.4)	1.32 (0.47–3.72)	
No	152 (77.6)	130 (88.5)	22 (14.5)		
Daily opioid^b^ injecting^a^					–
Yes	106 (53.8)	95 (89.6)	11 (10.4)	1.84 (0.81–4.20)	
No	91 (46.2)	75 (82.4)	16 (17.6)		
Daily crystal meth injecting^a^					
Yes	70 (35.5)	64 (91.4)	6 (8.6)	2.11 (0.81–5.51)	–
No	127 (64.5)	106 (83.5)	21 (16.5)		
Ever OD?					–
Yes	48 (24.7)	42 (87.5)	6 (12.5)	1.18 (0.45–3.11)	
No	146 (75.3)	125 (85.6)	21 (14.4)		
Drug treatment history					–
Yes	83 (42.8)	73 (88.0)	10 (12.0)	1.32 (0.57–3.05)	
No	111 (57.2)	94 (84.7)	17 (15.3)		

*p < 0.05

^a^In the past 6 months

^b^Opioids include heroin, methadone (prescribed and non-prescribed), Hydros (Dilaudid and Hydromorph Contin), generic oxycodone, Oxy Neo, percocet, and fentanyl

A total of 170 (86%) reported willingness to use SIS. In bivariable analyses (Table 1), those who expressed willingness to use SIS were less likely to be female (odds ratio (OR) 0.25; 95% confidence interval (CI) 0.11–0.60) and more likely to report any public injecting in the past 6 months (OR 3.61; 95% CI 1.55–8.44). In multivariable analyses, being female remained negatively associated with willingness to use SIS (adjusted odds ratio (AOR) 0.29; 95% CI 0.11–0.75), while public injecting in the past 6 months remained positively associated (AOR: 2.76; 95% CI: 1.00–7.62). Given our finding regarding gender, we tested all potential two-way interactions (where sufficient counts were available). No two-way interaction effects were found.

Among those who reported willingness to use, 106 (63%) said they would always/usually use SIS, while 62 (37%) said they would sometimes/occasionally use SIS (Table 2). Higher proportions of participants living in unstable housing (69 vs. 55%), and reporting any public injecting (68 vs. 50%), daily opioid injecting (71 vs. 50%) and daily crystal methamphetamine injecting (77 vs. 55%) in the past 6 months, reported higher expected frequency of SIS use if services were available (all *p <* 0.05).

**Table 2:** Demographic, drug use characteristics, and treatment history characteristics associated with expected frequency of use among PWID willing to use SIS

Characteristic	Expected frequency of SIS use		*p* value^b^
	Always or usually (*n =* 106) *n* (%)	Sometimes or occasionally (*n =* 62) *n* (%)	
Gender			
Female	35 (62.5)	21 (37.5)	0.9100
Male	71 (63.4)	41 (36.6)	
Ethnicity			
White	78 (61.4)	49 (38.5)	0.4276
Other	28 (68.3)	13 (31.7)	
Housing			
Unstable	70 (68.6)	32 (31.4)	0.0447*
Stable	36 (54.6)	30 (45.4)	
Sex work			
Yes	17 (53.1)	15 (46.9)	0.1939
No	89 (65.4)	47 (34.6)	
Any public injecting^a^			
Yes	85 (67.5)	41 (32.5)	0.0460*
No	20 (50.0)	20 (50.0)	
Any injecting alone^a^			
Yes	94 (63.1)	55 (36.9)	0.9952
No	12 (63.2)	7 (36.8)	
Any help injecting^a^			
Yes	35 (64.8)	19 (35.2)	0.7506
No	71 (62.3)	43 (37.7)	
Syringe sharing^a^			
Yes	27 (69.2)	12 (30.8)	0.3480
No	78 (60.9)	50 (39.1)	
Daily opioid^c^ injecting^a^			
Yes	74 (71.2)	30 (28.9)	0.0058*
No	32 (50.0)	32 (50.0)	
Daily crystal meth injecting^a^			
Yes	49 (76.6)	15 (23.4)	0.0045*
No	57 (54.8)	47 (45.2)	
Ever OD?			
Yes	30 (71.4)	12 (28.6)	0.1916
No	74 (60.2)	49 (39.9)	
Drug treatment history			
Yes	44 (62.0)	27 (38.0)	0.8066
No	60 (63.8)	34 (36.2)	

*p < 0.05

^a^In the past 6 months

^b^Determined through chi-square tests or Fishers exact, where appropriate

^c^Opioids include heroin, methadone (prescribed and non-prescribed), Hydros (Dilaudid and Hydromorph Contin), generic oxycodone, Oxy Neo, percocet, and fentanyl

Design preferences and the top ten amenities deemed important are described in Table 3. Approximately 84% of those who reported willingness to use SIS preferred private cubicles as set-up for injecting spaces, 82% would be willing to use an integrated service, located in a community health centre, hospital, doctor’s clinic, or social service agency, and 73% preferred daytime hours (8 am to 4 pm) for operation of services. Forty-nine percent reported that PWID should be involved in operating the service. No differences in design preferences were found by expected frequency of use (data not shown). The most important amenities identified for SIS include distribution of sterile injecting equipment, preventing and responding to overdoses, needle distribution, HIV/HCV testing, and washrooms (see Table 3 for top 10).

**Table 3:** Design preferences and important amenities identified for SIS among PWID willing to use SIS

Design feature	Percent
Willing to walk to SIS	88
Time willing to walk in the summer months	
≤20 min	60
>20 min	40
Time willing to walk in the winter months	
≤20 min	84
>20 min	16
Willing to take a bus to SIS	60
Time willing to take a bus in the summer months	
≤20 min	47
>20 min	53
Time willing to take a bus in the winter months	
≤20 min	54
>20 min	46
Willingness to use an integrated SIS	82
Preferred set-up for injecting space	
Private cubicle	84
An open plan with benches at one large table or counter	1
An open plan with tables and chairs	9
A combinatory of above	6
Preferred operating hours	
Daytime	73
Evening	20
Overnight	7
Involvement of PWID in SIS operation	49
Important amenities identified for SIS	Percent
Distribution of sterile injection equipment	98
Preventing and responding to overdoses	98
Needle distribution	97
HIV/HCV testing	96
Washrooms	94
Referrals to drug treatment, rehab and other services, when ready	94
Nursing staff for medical care and supervised injecting teaching	93
Access to health services	92
Harm reduction education	89
Withdrawal management	87

## Discussion

This study found high levels of willingness to use SIS among PWID in the mid-size city of London, Canada. Willingness to use SIS was positively associated with public injecting in the past 6 months and negatively associated with being female. Among those who were willing to use SIS, we found that participants living in unstable housing, those injecting in public, and those injecting opioids and crystal methamphetamine daily reported higher expected frequency of SIS use. We also characterized important considerations for implementing SIS locally. These findings have implications for maximizing the uptake and full potential of SIS in London, particularly among vulnerable groups of PWID.

While there are currently only two legally sanctioned supervised injection facilities established in North America [39, 40], both in Vancouver, numerous cities have conducted feasibility studies to determine the acceptability of these services among PWID, and to inform design and operational preferences. High levels of willingness to use SIS have generally been found across various large urban centres. A study conducted among an established cohort of PWID who were followed for 1 year between 2001 and 2002 in Vancouver found that 37% of PWID expressed willingness to use SIS [26]. However, a later study conducted in 2003 by the same authors among a different group of injection drug users in Vancouver found 92% willingness to use such a service [25]. Authors attributed the higher proportion of willingness in the latter study to the study’s participants who were active street-based injectors originating the centre of Vancouver’s open drug scene, the Downtown Eastside [25]. In more recent studies, willingness to use a safer injecting facility in San Francisco was 85% among PWID [23], while in Toronto and Ottawa, up to 75% of people who use drugs said they would use such a facility, if it were available [24]. Findings from the present study reveal that high levels of willingness to use SIS (86%), comparable to large urban centres, may also be found among PWID living in mid-sized cities.

High proportions (72%) of PWID in London reported injecting in public or semi-public spaces. Consistent with previous feasibility studies conducted [23, 24], we also found that public injecting was associated willingness to use SIS. Public injecting poses risks to individual health through rushed injection practices and reduced ability to ensure privacy, safety and hygiene [41–43]. It is also associated with elevated risk of syringe sharing, blood-borne infections, and overdose [43, 44]. Interestingly, among those who expressed willingness to use SIS, those who reported higher expected frequency of SIS use represent more vulnerable groups of PWID, such as those who live in unstable housing, and those reporting public injecting and daily opioid or crystal methamphetamine injection. This finding has significant implications, suggesting that those who are especially vulnerable to adverse health outcomes would more frequently use the service, if available. Similar findings have been reported in Vancouver, where SIFs were found to attract those who injected in public, those who were homeless or unstably housed, and those who injected heroin daily [45]. Accordingly, SIS may be effective in attracting and connecting vulnerable groups of PWID to medical care, access to clean injecting equipment, emergency response to drug overdose, and referrals to addiction treatment and other support services. Likewise, given the association between public injecting and homelessness, SIS present the opportunity to link PWID to housing programmes, including “Housing First” initiatives that offer a recovery-oriented approach centred on providing independent and permanent housing to individuals experiencing homelessness [46]. Therefore, SIS in London can potentially reduce the harms associated with injection drug use, and more specifically, public injecting. At the same time, given that public injecting contributes to the improper disposal of injection-related litter, SIS can also improve public order through reduced numbers of publicly discarded syringes and injection-related litter [7]. In this way, our findings highlight not only the potential for SIS to impact PWID but also broader communities where PWID live and inject.

In the context of injection drug use, sex and gender act as determinants of health, shaping risk profiles between men and women and contributing to unique barriers to treatment and access to services [47–49]. We found that women were less likely to express willingness to use SIS. One possible explanation of this finding could be that compared to men, women who use illicit drugs experience greater stigmatization, which acts as a barrier to seeking care, including access to addiction treatment and harm reduction services [50–52]. Feelings of guilt and shame and lower levels of self-esteem and self-efficacy resulting from stigma, in addition to experiences of violence related to drug use can prevent women from seeking help or feeling like they deserved to be helped [50, 51, 53]. Some women may also be hesitant to publicly access services which may result in the disclosure of their drug use and exacerbate experiences of stigma.

Given our findings, gender considerations should inform the design and implementation of SIS to ensure equitable access and uptake, especially in light of past work indicating that SIS can provide refuge from gendered violence in local drug scenes [53, 54]. Tailored harm reduction approaches for women may include a women’s-only SIS, women’s specific drop-in times, and women-centred health and social service programming, and case management teams [49, 55, 56].

Lower rates of willingness to use SIS among women in this setting stands out in contrast to other SIS feasibility work undertaken in large urban centres [23, 24] and past research showing similar rates of actual SIS uptake across genders [57]. Future research should seek to explore these differences. However, it should be noted that while in our multivariable model women appear less likely to express willingness to use SIS, the raw values still express a high willingness (76% for women, 93% for men).

This research also provided important information on the design and operational preferences of PWID in London that can inform the implementation of SIS. Among those who were willing to use SIS, most were willing to walk or take the bus to access the service. Interestingly, we found that fewer PWID were willing to walk longer (i.e. greater than 20 min) in winter months compared to summer months (16% compared to 40%). However, when it came to taking a bus, there was less difference in willingness to take bus in terms of time between winter and summer months (46% compared to 53%). This is somewhat in contrast to work undertaken elsewhere, which found that PWID were not generally willing to travel distances to use a SIS [58]. Further, most PWID preferred private cubicles for injecting and daytime service hours, while just under half believed PWID should be involved in the operation of services. With regard to private cubicles, this may reflect PWIDs’ awareness of Insite, Canada’s first sanctioned SIS, which uses private cubicles, or may reflect the needs of some, in particular women, to ensure privacy and control over their drugs and drug use [53]. Important amenities identified for SIS in this setting include distribution of sterile needles and other injection equipment, preventing and responding to overdoses, access to HIV and HCV testing, availability of washrooms, access to health services, referrals to drug treatment and other support services, harm reduction education and withdrawal management. Similar important services to provide alongside SIS were found in feasibility work done in Ottawa, but not in Toronto, where access to nursing and medical staff, food, drug counsellors and urgent detox beds were identified, highlighting the distinct preferences of PWID in each setting [24].

Given the current opioid and overdose epidemics experienced across North America, the findings from the present study are timely and have implications for other small or mid-sized cities that are dealing with a disproportionately high burden of injection drug use. The present study suggests that PWID of mid-sized cities are overwhelmingly willing to use SIS, taking into consideration distinct design and operational preferences.

Further, the findings of the present study, in line with the outcomes of a recent study from London that found overall community stakeholder support of SIS implementation [59], challenge past research suggesting that in contrast to large urban centres, all smaller cities lack liberal perspectives toward harm reduction and injection drug use [31].

There were limitations in this study. Participants recruited were not randomly sampled, and therefore may not be representative of all PWID in London. Although efforts were made to recruit participants from a diverse range of settings, this may have resulted in some group or social networks being over-represented in our sample. We also relied on self-reported data collected by peer research associates, which may be subject to response bias, including social desirability and recall bias. However, it is worth noting that self-reported responses from PWID can be valid and reliable [60].

## Conclusions

In conclusion, a high proportion of PWID in London were willing to use SIS if services were available, with willingness to use being positively associated with public injecting and negatively associated with being female. Likewise, among those expressing willingness to use SIS, those who reported higher expected frequency of SIS use represented more vulnerable groups of PWID. These findings suggest that SIS may play a role in addressing the harms associated with injection drug use, particularly among vulnerable groups of PWID. With high levels of SIS acceptability among PWID and London’s Board of Health recently approving to move forward with exploring the implementation of SIS [61], next steps for London include the consultation of a diverse range of stakeholders and the broader community, including the City of London, the Middlesex-London Health Unit, London Police Service, local businesses, and residents. Nonetheless if implemented, to maximize the uptake and potential benefits of SIS, policy makers and programme planners should take into the consideration the attributes and preferences of local PWID.

## Abbreviations

AOR: Adjusted odds ratio
CI: Confidence interval
HCV: Hepatitis C virus
HIV: Human immunodeficiency virus
OR: Odds ratio
PRA: Peer research associate
PWID: People who inject drugs
SIF: Safe injection facility
SIS: Supervised injection services
